# Influenza Vaccination Is Associated With Lower Incidental Asthma Risk in Patients With Atopic Dermatitis: A Nationwide Cohort Study

**DOI:** 10.3389/fimmu.2021.729501

**Published:** 2021-10-12

**Authors:** Kun Hong Li, Pui-Ying Leong, Chung-Fang Tseng, Yu Hsun Wang, James Cheng-Chung Wei

**Affiliations:** ^1^ Institute of Medicine, Chung Shan Medical University, Taichung, Taiwan; ^2^ Department of Family Medicine, Changhua Christian Hospital, Changhua, Taiwan; ^3^ Department of Medicine, Chung Shan Medical University Hospital, Taichung, Taiwan; ^4^ Division of Allergy, Immunology and Rheumatology, Chung Shan Medical University Hospital, Taichung, Taiwan; ^5^ Department of Medical Research, Chung Shan Medical University Hospital, Taichung, Taiwan; ^6^ Graduate Institute of Integrated Medicine, China Medical University, Taichung, Taiwan

**Keywords:** asthma, atopic dermatitis, big data analysis, influenza vaccination, Taiwan national health insurance research database

## Abstract

**Background:**

Atopic march refers to the natural history of atopic dermatitis (AD) in infancy followed by subsequent allergic rhinitis and asthma in later life. Respiratory viruses interact with allergic sensitization to promote recurrent wheezing and the development of asthma. We aimed to evaluate whether influenza vaccination reduces asthma risk in people with AD.

**Methods:**

This cohort study was conducted retrospectively from 2000 to 2013 by the National Health Insurance Research Database (NHIRD). Patients with newly diagnosed AD (International Classification of Diseases, Ninth Revision, Clinical Modification code 691) were enrolled as the AD cohort. We matched each vaccinated patient with one non-vaccinated patient according to age and sex. We observed each participant until their first asthma event, or the end of the study on December 31, 2013, whichever came first.

**Results:**

Our analyses included 4,414 people with a mean age of 53 years. Of these, 43.8 were male. The incidence density of asthma was 12.6 per 1,000 person-years for vaccinated patients, and 15.1 per 1000 person-years for non-vaccinated patients. The adjusted hazard ratio (aHR) of asthma in the vaccinated cohort relative to the non-vaccinated cohort was 0.69 (95% CI = 0.55–0.87). Vaccinated patients had a lower cumulative incidence of asthma than unvaccinated patients. Vaccinated participants in all age and sex groups trended toward a lower risk of asthma. People will reduce more asthma risk when taking shots every year.

**Conclusion:**

Influenza vaccination was associated with lower asthma risk in patients with AD.

## Introduction

Up to 80% of children with atopic dermatitis (AD) develop asthma or allergic rhinitis later in life (1). Teenagers with asthma have higher AD rates than those without asthma (risk ratio 4.5, 95% CI = 3.1–6.5) (2). The most common mechanisms for this occurrence are barrier defects, skin inflammation, and microbiome alterations that trigger the T‐helper type 2 (Th2) cell response and lead to hypersensitization for later disorders. AD and asthma share similar genetic loci, and people afflicted by these conditions often share similar food allergies and early environmental exposures. Vitamin D, probiotics, allergen avoidance, allergen immunotherapy, IgE antagonists, and respiratory infection prevention are all considered strategies for asthma prevention (3). Viral respiratory tract infections in infancy, particularly influenza virus (4–6), respiratory syncytial virus, and human rhinovirus may predict asthma development from late childhood through early adulthood (7). Respiratory viruses interact with allergic sensitization and other microbes to promote recurrent virus- induced wheezing and asthma development *via* a number of mechanisms, including increased inflammatory cell recruitment, promotion of cytokine production, enhanced allergic inflammation, and augmented airway hyperresponsiveness (8).

Children and adults with asthma have increased risks of hospitalization and respiratory morbidity due to acute influenza respiratory infections (9). Children with asthma were particularly prone to increased intensive care unit (ICU) stays and pneumonia during the 2009 H1N1 influenza pandemic. Influenza is also associated with a higher risk of emergency department (ED) treatment failure for acute respiratory illness (10).

To protect asthmatic patients, annual influenza vaccination is recommended by the Advisory Committee for Immunization Practices (ACIP), the American Academy of Pediatrics (AAP), and the Expert Panel for the Diagnosis and Management of Asthma. A recent systemic review and meta- analysis including observational studies suggested that influenza vaccination reduced the risk of asthma’s exacerbation (11).

However, the association between influenza vaccination and further asthma risk has not yet been explored, particularly in patients with AD. We conducted an original longitudinal nationwide cohort study to determine whether influenza vaccination in AD patients decreases the risk of asthma.

## Materials and Methods

### Data Source

We analyzed anonymous data from the Taiwan National Health Insurance Research Database (NHIRD). The Taiwan National Health Insurance (NHI) program launched in 1995 and covered 99% of Taiwanese residents. The comprehensive claims data from the NHI program were collected into NHIRD. We utilized the outpatient and inpatient records of one million randomly sampled people in the Longitudinal Health Insurance Database. There were no statistically significant differences in sex, age, or healthcare costs between the sample group and all enrollees. The database used the International Classification of Diseases, Ninth Revision, Clinical Modification (ICD-9-CM) codes to define the patients’ corresponding diseases. Due to safety and privacy concerns, all patient identification information was encrypted.

### Study Population

This cohort study was conducted retrospectively from 2000 to 2013. Newly diagnosed AD patients (ICD-9-CM = 691) with at least three outpatient visits or one inpatient admission record were enrolled as the AD cohort. The inclusion criteria were defined as people who received an influenza vaccine after AD diagnosis and before January 1, 2013. The earliest influenza vaccination date was used as the index date for the vaccinated group. We excluded people with a history of asthma (ICD-9-CM = 493) prior to the index date. Patients who never received an influenza vaccination after AD diagnosis were enrolled as the non-vaccinated cohort. The index date of the unvaccinated group was assigned by 1:1 age and sex matching. We observed each participant until the first asthma event or the end of the study on December 31, 2013, whichever came first. This study was approved by the NHIRD research committee and the Joint Institutional Review Board of Chung Shan Medical University (IRB permit number: CS19009).

### Main Explanatory Variable

The main explanatory variable in this study was influenza vaccination, which was identified using the therapeutic treatment code A2001C, the ICD-9-CM codes V04.7 or V04.8, or the medication codes for influenza vaccination. The flu usually strikes between November and the following March. In Taiwan, influenza vaccination has been free for people with high-risk comorbidities since 2001, and for all adults over 65 since 1998. This was extended to infants and children between the ages of 6 months and 2 years in 2004, with gradual extension to fifth- and sixth-grade–aged elementary school students in 2012. The vaccines in the influenza immunization program were Adimflu-S (ADIMMUNE Corporation, Taiwan); Fluvirin (Novartis Vaccines, Switzerland); AGRIPPAL S1 (Novartis Vaccines, Switzerland); Begrivac (Novartis Vaccines, Switzerland); Vaxigrip (Pasteur Merieux Connaught, France); and Fluarix (GlaxoSmithKline, USA). Each influenza vaccination program recorded the influenza vaccination status of all vaccinated participants.

### Main Outcome and Comorbidities

Asthma was the primary endpoint in this study. We defined asthma patients as patients with three or more outpatient visits or one inpatient record of ICD-9-CM code 493. The following comorbidities developed before the index date were considered as confounders (ICD-9-CM codes indicated in parentheses): hypertension (401–405); hyperlipidemia (272.0–272.4); chronic liver disease (571); chronic kidney disease (585); diabetes (250); gastroesophageal reflux disease (530.11, 530.81, 530.85); allergic rhinitis (472, 473, 477); urticaria (708); chronic obstructive pulmonary disease (COPD; 491, 492, 496); obstructive sleep apnea (327.23, 780.51, 780.52, 780.53, 780.57); cellulitis (682); attention deficit hyperactivity disorder (314.0); anxiety (300.0); and depression (296.2, 296.3, 300.4, 309.0, 309.1, 309.28, 311).

### Statistical Analysis

The data were computed as percentages for categorical variables, and as means for continuous variables. We examined the baseline variables of the vaccinated and non-vaccinated cohorts using chi-square, Fisher’s exact, and Student’s t-tests. A Kaplan–Meier analysis was used to acquire the cumulative incidence curve of each cohort.

We applied the Cox proportional hazards model to estimate asthma’s hazard ratio. We also performed a subgroup analysis of the association between influenza vaccination and asthma development. Finally, we performed a sensitivity test to explore the effect of time on this study. All statistical analyses were conducted with SPSS version 18.0 (SPSS Inc., Chicago, IL, USA). P-values of less than 0.05 were considered significant.

## Results

**Figure 1:**
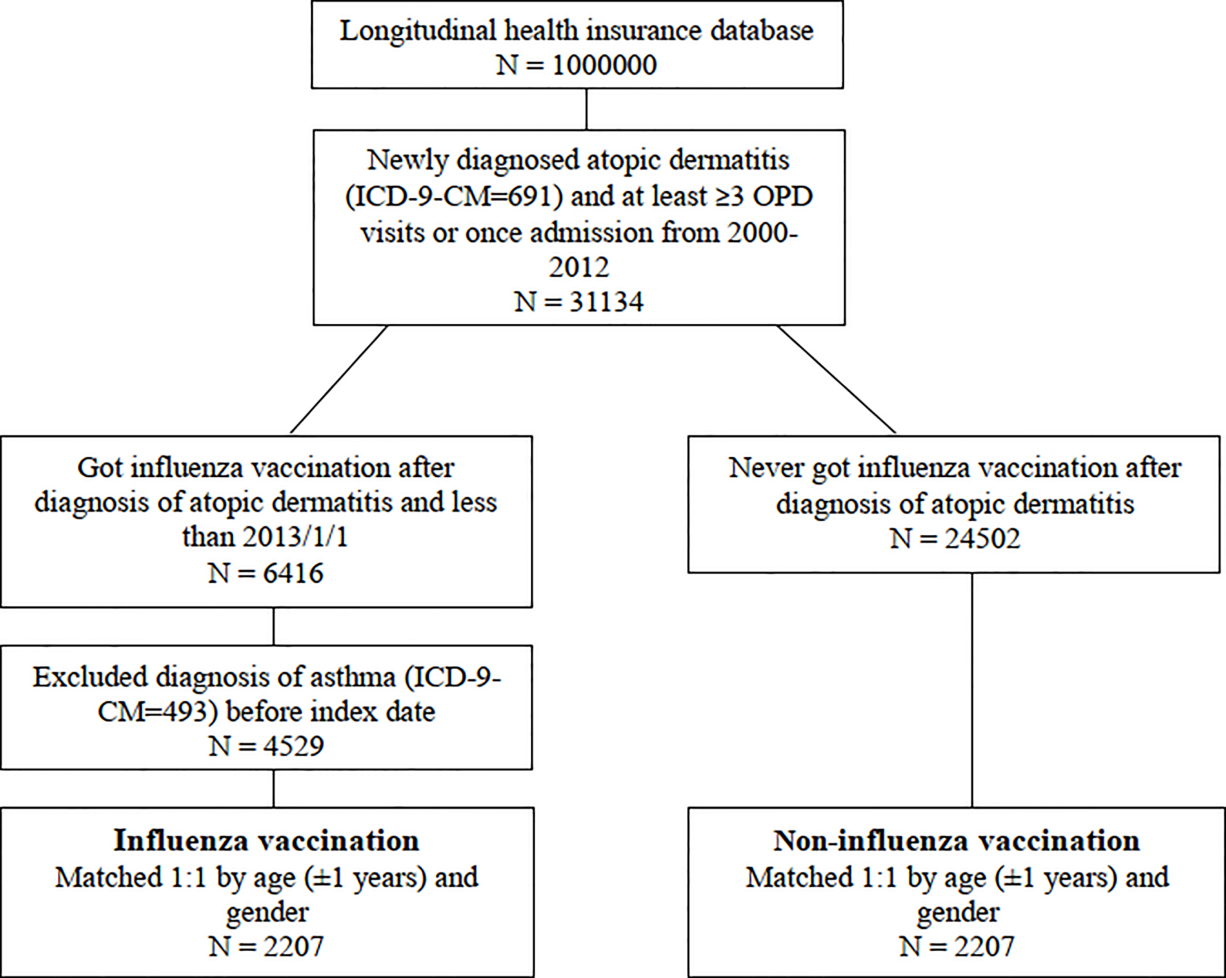
Flowchart of subject's enrollment with and without influenza vaccination.

This study identified 31,134 people in Taiwan who had newly diagnosed AD (**Figure 1**). During the follow-up period, 20.6% (6,416 people) received an influenza vaccination. After excluding 1,887 people with antecedent asthma, we included the remaining 2,207 people with and without vaccination in each group after sex and age matching in our analyses (**Table 1**).

**Table 1:** Demographic characteristics of influenza vaccination group and non-influenza vaccination.

	Influenza vaccination (N = 2207)	Non-influenza vaccination (N = 2207)	p value
Age			<0.001
<20	146 (6.6)	151 (6.8)	
20-39	411 (18.6)	418 (18.9)	
40-64	752 (34.1)	948 (43.0)	
≥65	898 (40.7)	690 (31.3)	
Mean ± SD	53.5 ± 19.1	53.1 ± 18.8	0.404
Gender			1.000
Female	1240 (56.2)	1240 (56.2)	
Male	967 (43.8)	967 (43.8)	
Hypertension	647 (29.3)	515 (23.3)	<0.001
Hyperlipidemia	235 (10.6)	194 (8.8)	0.037
Chronic liver disease	118 (5.3)	84 (3.8)	0.014
Chronic kidney disease	45 (2.0)	12 (0.5)	<0.001
Diabetes	364 (16.5)	263 (11.9)	<0.001
GERD	49 (2.2)	34 (1.5)	0.096
Allergic rhinitis	166 (7.5)	144 (6.5)	0.195
Urticaria	139 (6.3)	97 (4.4)	0.005
COPD	80 (3.6)	40 (1.8)	<0.001
OSA	100 (4.5)	79 (3.6)	0.109
Cellulitis	68 (3.1)	44 (2.0)	0.022
ADHD	0 (0.0)	2 (0.1)	0.500^†^
Anxiety	107 (4.8)	105 (4.8)	0.888
Depression	96 (4.3)	54 (2.4)	<0.001

^†^Fisher’s exact test

COPD, Chronic obstructive pulmonary disease.

OSA, Obstructive sleep apnea.

ADHD, attention-deficit hyperactivity disorder.

The majority (74%) of our study’s participants were over 40 years old. The mean age was 53 years old. Of the total number of participants, 43.8% were male and 56.2% were female. Vaccinated participants had a slightly higher proportion of concurrent conditions than did unvaccinated participants, including hypertension, chronic kidney disease, diabetes, COPD, and depression.

The asthma incidence density of vaccinated participants was 12.6 per 1,000 person-years, whereas that of non-vaccinated participants was 15.1 per 1,000 person-years (**Table 2**). The adjusted hazard ratio (aHR) of asthma for the vaccinated cohort relative to the non-vaccinated cohort was 0.69 (95% CI = 0.55–0.87). Allergic rhinitis and COPD increased the risk of asthma in participants by 2.37-fold (95% CI = 1.70–3.31) and 2.53-fold (95% CI = 1.62–3.94), respectively.

**Table 2:** Cox proportional hazard model analysis for risk of asthma.

	No. of asthma	PY	ID	Univariate		Multivariate	
				HR (95% C.I.)	p value	HR (95% C.I.)	p value
Group							
Non-influenza vaccination	172	11406	15.1	1		1	
Influenza vaccination	134	10639	12.6	0.81 (0.65-1.02)	0.073	0.69 (0.55-0.87)	0.002
Age							
<20	20	1463	13.7	1		1	
20-39	40	3645	11.0	0.76 (0.45-1.30)	0.323	0.77 (0.45-1.33)	0.351
40-64	92	8259	11.1	0.80 (0.49-1.30)	0.371	0.75 (0.46-1.24)	0.263
≥65	154	8677	17.7	1.34 (0.84-2.14)	0.222	1.26 (0.77-2.06)	0.360
Gender							
Female	190	12221	15.5	1		1	
Male	116	9824	11.8	0.77 (0.61-0.97)	0.024	0.75 (0.59-0.95)	0.015
Hypertension	101	5606	18.0	1.42 (1.12-1.81)	0.004	1.15 (0.87-1.51)	0.321
Hyperlipidemia	29	1903	15.2	1.06 (0.72-1.56)	0.759	0.87 (0.58-1.30)	0.495
Chronic liver disease	11	967	11.4	0.80 (0.44-1.45)	0.458	0.72 (0.39-1.32)	0.283
Chronic kidney disease	2	214	9.3	0.60 (0.15-2.43)	0.477	0.61 (0.15-2.48)	0.490
Diabetes	51	3012	16.9	1.25 (0.92-1.69)	0.148	1.12 (0.81-1.56)	0.494
GERD	9	269	33.4	2.13 (1.09-4.13)	0.026	1.78 (0.91-3.50)	0.095
Allergic rhinitis	45	1323	34.0	2.59 (1.89-3.56)	<0.001	2.37 (1.70-3.31)	<0.001
Urticaria	25	1082	23.1	1.68 (1.12-2.53)	0.013	1.51 (1.00-2.29)	0.052
COPD	24	480	50.0	3.64 (2.40-5.52)	<0.001	2.53 (1.62-3.94)	<0.001
OSA	21	801	26.2	1.87 (1.20-2.92)	0.006	1.34 (0.83-2.18)	0.233
Cellulitis	8	557	14.4	1.02 (0.51-2.07)	0.947	0.86 (0.42-1.74)	0.676
Anxiety	27	989	27.3	2.03 (1.36-3.01)	<0.001	1.46 (0.94-2.26)	0.096
Depression	17	624	27.3	1.92 (1.18-3.13)	0.009	1.36 (0.78-2.36)	0.273

ID, Incidence density (per 1000 person-years).

PY, person-years.

COPD, Chronic obstructive pulmonary disease.

OSA, Obstructive sleep apnea.

Multivariate, adjusted for age, gender, hypertension, hyperlipidemia, chronic liver disease, chronic kidney disease, diabetes, GERD, allergic rhinitis, urticaria, COPD, OSA, cellulitis, anxiety, and depression.

**Figure 2:**
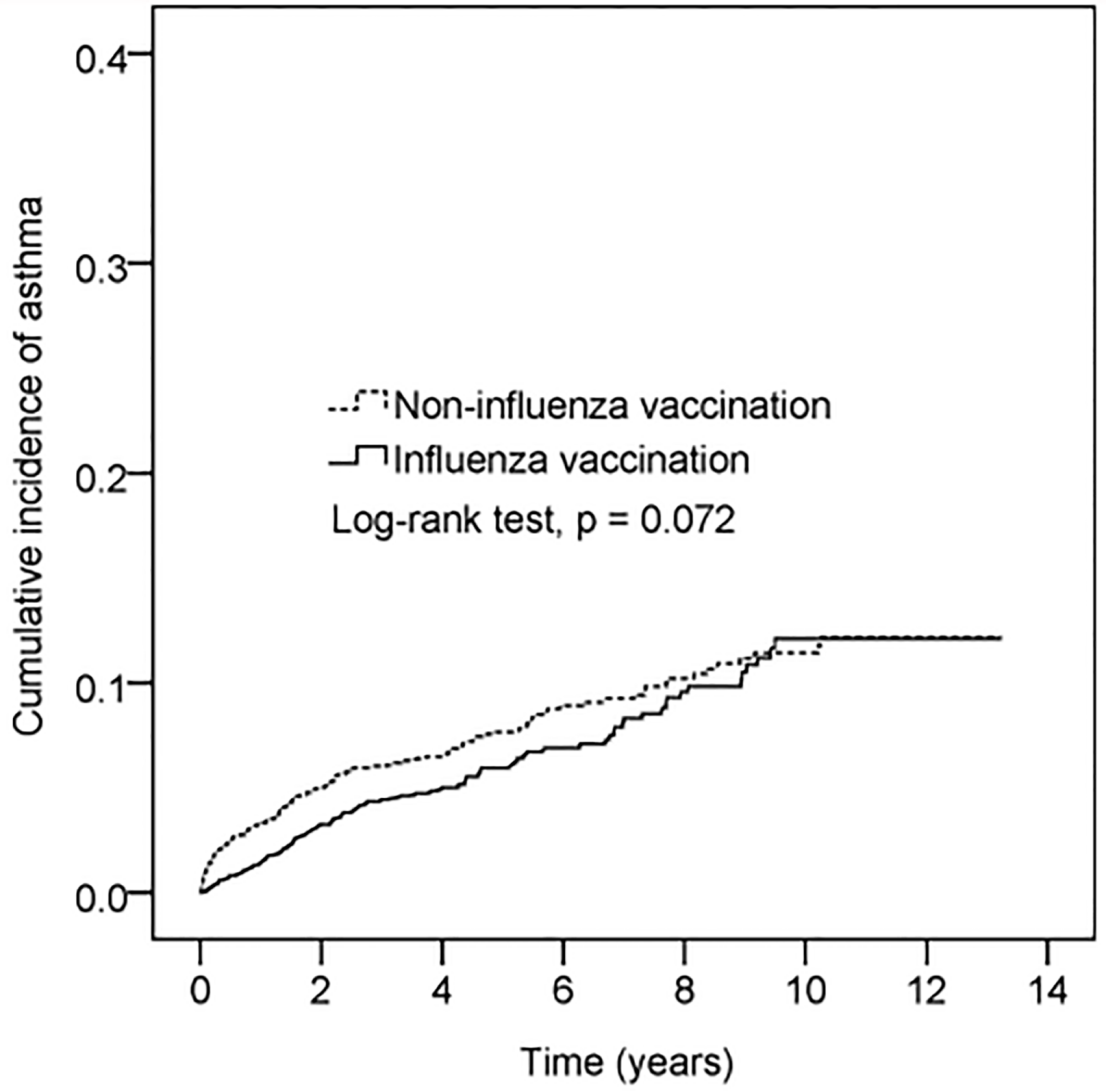
Cumulative incidence of asthma in patients with vaccination *vs* without vaccination. The x-axis represents years after flu vaccination. The index date of the unvaccinated group was assigned by 1:1 age and sex matching. Y-axis represents cumulative incidence of asthma.

The cumulative incidence of asthma was lower in vaccinated participants than in non-vaccinated participants (**Figure 2**). However, this difference was not statistically significant (log-rank test p = 0.072).

**Table 3** presents the stratification analysis of the association between influenza vaccination and asthma by age and sex. Vaccinated participants in all age groups trended toward a lower risk of asthma (hazard ratio (HR) = 0.54–0.94), though this trend was not statistically significant. Influenza vaccination did not significantly reduce the risk of asthma in men or women; this was attributed to the small sample size. However, a decreasing trend was identified clinically in both men and women, with HR of 0.76 and 0.90, respectively.

**Table 3:** Subgroup analysis of the association between influenza vaccination and asthma development.

	Influenza vaccination		Non-influenza vaccination		HR (95% C.I.)	p value
	N	No. of asthma	N	No. of asthma		
Age						
<20	146	7	151	13	0.54 (0.22-1.36)	0.190
20-39	411	15	418	25	0.60 (0.32-1.14)	0.117
40-64	752	32	948	60	0.69 (0.45-1.06)	0.092
≥65	898	80	690	74	0.94 (0.68-1.29)	0.685
p for interaction = 0.434						
Gender						
Female	1240	81	1240	109	0.76 (0.57-1.01)	0.057
Male	967	53	967	63	0.90 (0.63-1.30)	0.588
p for interaction = 0.433						

We conducted sensitivity analyses to see if the associations of influenza vaccination and incident asthma affected by different follow up duration (**Table 4**). Influenza vaccination significantly and consistently reduced the risk of incident asthma in the last three to seven years of the study.

**Table 4:** Sensitivity analysis of the association between influenza vaccination and asthma development.

	N	No. of asthma	Univariate		Multivariate	
			HR (95% C.I.)	p value	HR (95% C.I.)	p value
Follow-up duration ≤3 years						
Group						
Non-influenza vaccination	2207	129	1		1	
Influenza vaccination	2207	92	0.71 (0.55-0.93)	0.013	0.61 (0.47-0.81)	<0.001
Follow-up duration ≤5 years						
Group						
Non-influenza vaccination	2207	149	1		1	
Influenza vaccination	2207	109	0.74 (0.58-0.95)	0.016	0.63 (0.49-0.81)	<0.001
Follow-up duration ≤7 years						
Group						
Non-influenza vaccination	2207	160	1		1	
Influenza vaccination	2207	122	0.78 (0.62-0.99)	0.040	0.66 (0.52-0.84)	<0.001

Multivariate: adjusted for age, gender, hypertension, hyperlipidemia, chronic liver disease, chronic kidney disease, diabetes, GERD, allergic rhinitis, urticaria, COPD, OSA, cellulitis, anxiety, and depression.

**Table 5** presented the asthma risk reduction is dose dependent. Compared with non-vaccinated group, people who received once flu vaccination didn’t have great effect on asthma reduction. But, for those more than 2 times, the HR was 0.59 (0.44-0.79). We inferred that people will reduce more asthma risk when taking shots every year.

**Table 5:** influenza vaccination dose response on asthma risk reduction.

	Number	No. of asthma	HR^†^ (95% C.I.)	p value
Group				
Non-influenza vaccination	2207	172	1	
Influenza vaccination =1 time	1151	63	0.91 (0.67-1.23)	0.535
Influenza vaccination ≥2 times	1056	71	0.59 (0.44-0.79)	<0.001

^†^Adjusted for age, gender, hypertension, hyperlipidemia, chronic liver disease, chronic kidney disease, diabetes, GERD, allergic rhinitis, urticaria, COPD, OSA, cellulitis, anxiety, and depression.

## Discussion

### Principal Findings

Our 13-year retrospective cohort study revealed a decreased risk of asthma in vaccinated AD patients with an aHR of 0.69 (95% CI = 0.55–0.87) after adjusting for the age, sex, and confounders listed in **Table 2**. Although our subgroup analysis did not yield significant results, we found that influenza vaccination reduced asthma development in all age and sex groups.

### Theoretical Mechanism

Atopic march, sometimes called allergic march, refers to AD’s natural history and typical progression in infancy, followed by subsequent allergic rhinitis and asthma in later childhood. We have analyzed Influenza infection times between vaccinated and non-vaccinated group. There are no significant infection times differences with and without Influenza vaccination throughout the study period. The mechanism may not be that influenza vaccination directly prevents and/or mitigates the severity of influenza infection, but immune modulation is favor. Allergic sensitization and bacterial colonization due to a dysfunctional skin barrier promote Th2 immunity, which induces systemic responses in the respiratory tract (12). On the other hand, the acute stage of influenza not only causes inflammation and tissue damage to the respiratory tract, but also enhances unrelated local allergic responses *via* the Th2 response (13). Accordingly, the Th2 subset may not play a primary role in virus clearance and recovery, and may lead to immune-mediated injury potentiation (14–16). One way to prevent subsequent asthma and atopic disorders is by using vaccination to restore the Th1/Th2 balance in favor of Th1. An animal study by Skevaki found that influenza-infected animals showed heterologous immunity toward allergens. Immunization *via* vaccination with influenza-derived peptides provided asthma protection through the interferon-gamma response (17). Another study showed influenza vaccination to activate CD4+ and Th1–type cells, which induced the secretion of Th1-type cytokines and promoted T cell immunity (18).

### Clinical Implications

An estimated 65%–80% of children with AD develop symptoms in their first year of life. Asthma has a later onset, occurring in only 42% of children in their first year. However, 92% of affected individuals develop symptoms by age 8 (19). Furthermore, patients who experienced asthma onset prior to 1 year of age were reported to have more severe symptoms and greater medical expenses than patients who developed asthma symptoms between 5 and 9 years of age (20). Therefore, efforts to delay early asthma onset will be essential to prevent atopic march and the subsequent outcome of asthma. As shown in the illustration in **Figure 2** and **Table 4**, influenza vaccination can delay the onset of asthma by at least seven years. According to the 2006 report by the International Study of Asthma and Allergies in Childhood (21), AD has a prevalence of 6.7% in 6-to-7-year-old Taiwanese children, but only 4.1% in 13-to-14-year-old Taiwanese children. Such findings indicate that age-related physiological changes may lessen the symptoms of AD or cause them to disappear spontaneously.

This study evaluated patients who received free influenza vaccinations that were provided in Taiwan to children, the elderly, and people with comorbidities. We found that the vaccinated group had an increased percentage of underlying chronic disease than did the non-vaccinated group. Because asthma is associated with hypertension, diabetes mellitus, dyslipidemia, and cardiovascular disease (22), this difference underestimates the protective effect of influenza vaccination on asthma development. Furthermore, in our stratification study investigating the association of asthma with other comorbidities, we found that asthma increased the risk of developing allergic rhinitis and COPD by 2.37-fold and 2.53-fold, respectively. Therefore, we conclude that people with allergic diseases may benefit from influenza vaccination.

### Strengths and Limitations

The primary strength of this study is that it included a long-term comprehensive follow-up from 2000–2013, with universal coverage for all age groups. Secondly, we found an excellent positive predictive value (90%–100%) in the inpatient setting, validating the ICD-9-CM code 691 for AD (23). We also qualified the definitions of asthma and AD in our study to include a minimum of three outpatient records or one inpatient record. Our database size ensured similar distributions in each group due to well-balanced matching, which reduced the study’s heterogeneity and selection bias. To further reduce bias, we performed a sensitivity analysis for unmeasured confounders. Importantly, this is the first population-based cohort study to show that influenza vaccination could reduce the incidence of asthma in AD patients.

There are some limitations to this study, particularly since there is no consensus on asthma’s diagnosis, especially in children. The ICD-9-CM code 493–based algorithm for ascertaining asthma had a sensitivity of 82% and specificity of 98% (24), and a positive predictive value of 75.0% when compared to the criteria-based medical record review (25). The ICD-9-CM code 493 is widely accepted for etiologic research in asthma, but may underestimate its prevalence. Further efforts are needed to check the consistency of diagnosis by medical chart review. In order to reduce confounding by indication, an active comparator (i.e., other vaccines) is needed to serve as a control group. Finally, the clinical relevance of this study must be further validated by larger-scale prospective randomized trials.

## Conclusion

This long-term nationwide cohort study revealed that influenza vaccination was associated with lower incidental asthma risk in people with AD after adjusting for age, gender, and comorbidities. Nevertheless, more comprehensive studies are needed to confirm our findings.

## Data Availability Statement

The original contributions presented in the study are included in the article/supplementary material. Further inquiries can be directed to the corresponding author.

## Author Contributions

All authors contributed to the article and approved the submitted version. Conception and design: KHL and JC-CW. Acquisition of data: YHW. Analysis and interpretation of data: KHL, YHW, and JC-CW. Writing (original draft preparation): KHL. Writing (review and editing): P-YL, C-FT, and JC-CW.

## Funding

This study was supported by Chung Shan Medical University Hospital (CSH-2019-C-004). The funders had no role in the design and conduct of the study; the collection, management, analysis, and interpretation of the data; or the preparation, review, or approval of the manuscript.

## Conflict of Interest

The authors declare that the research was conducted in the absence of any commercial or financial relationships that could be construed as a potential conflict of interest.

## Publisher’s Note

All claims expressed in this article are solely those of the authors and do not necessarily represent those of their affiliated organizations, or those of the publisher, the editors and the reviewers. Any product that may be evaluated in this article, or claim that may be made by its manufacturer, is not guaranteed or endorsed by the publisher.
